# Network modeling predicts how DYRK1A inhibition promotes cardiomyocyte cycling after ischemia/reperfusion injury

**DOI:** 10.1172/jci.insight.200429

**Published:** 2026-09-17

**Authors:** Bryce C. Murillo, Alexander Young, Kaitlyn L. Wintruba, Alexander J. Eichert, Klara Siejda, Dennon Hoernig, Leigh A. Bradley, Bryana N. Harris, Catherine Zhao, Michelle Wu, Emmanuel Deau, Mattias F. Lindberg, Laurent Meijer, Jeffrey J. Saucerman, Matthew J. Wolf

**Affiliations:** 1 Department of Pharmacology,; 2 Department of Medicine,; 3 Department of Biomedical Engineering, and; 4 Department of Molecular Physiology and Biological Physics, University of Virginia, Charlottesville, Virginia, USA.; 5 Perha Pharmaceuticals, Roscoff, France.

**Keywords:** Cardiology, Cell biology, Cardiovascular disease, Cell cycle, Mouse models

## Abstract

The adult mammalian heart has a limited ability to regenerate lost myocardium following myocardial infarction (MI), largely due to the poor proliferative capacity of cardiomyocytes (CMs). Dual-specificity tyrosine phosphorylation–regulated kinase 1A (DYRK1A) is a known regulator of cell quiescence, though the mechanisms underlying its function remain unclear. Previous studies have shown that pharmacological inhibition of DYRK1A using harmine induces CM cell cycle reentry after ischemia/reperfusion (I/R) MI. Here, we developed a computational network model of DYRK1A-mediated regulation of the cell cycle, which predicts how DYRK1A inhibition promotes CM reentry. To validate these predictions, we tested selective DYRK1A inhibitors and observed robust induction of cell cycle activity in neonatal rat cardiomyocytes (NRCMs). Integrating our network model with bulk RNA sequencing data from DYRK1A inhibitor–treated NRCMs, we identified E2F1 as a key transcriptional driver of cell cycle gene expression. Finally, we demonstrate that both pharmacological and post-developmental inhibition of DYRK1A enhances heart function and increases CM cycling following I/R MI. Our findings suggest that functional recovery induced by small-molecule inhibitors of DYRK1A is mediated by the induction of cycling CMs.

## Graphical abstract

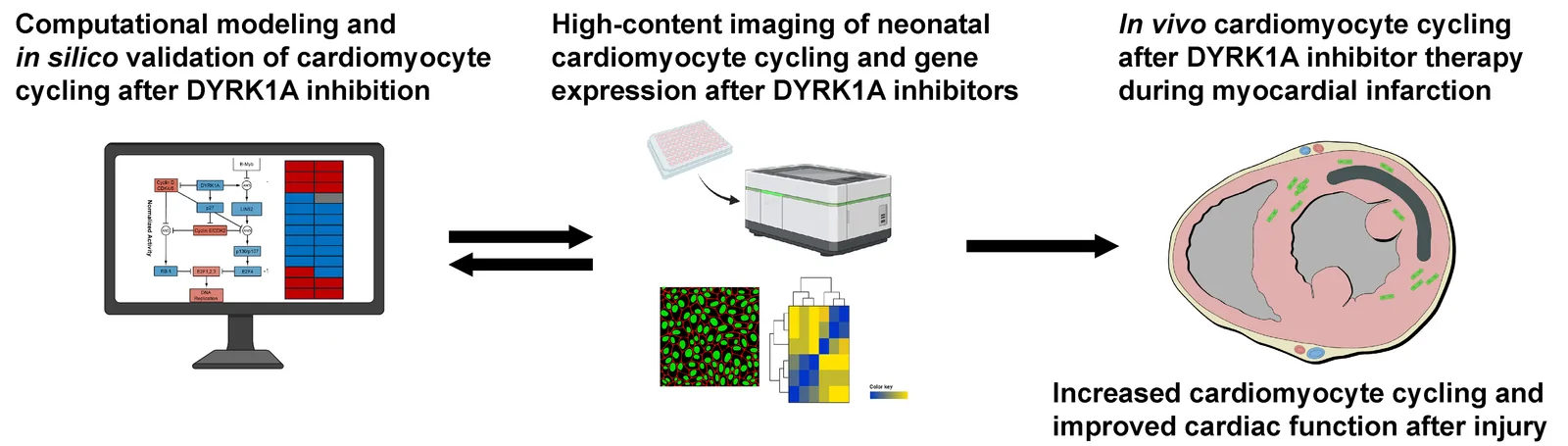

## Introduction

Myocardial infarctions (MIs) affect approximately 800,000 individuals in the United States annually and cause significant morbidity and mortality (1). The ischemia induced by the cessation of coronary blood flow during an MI and subsequent reperfusion lead to cardiomyocyte (CM) necrosis, infiltration of inflammatory cells, and activation of fibroblasts to produce fibrosis (2). Unfortunately, the adult mammalian heart has a limited regenerative capacity and is unable to adequately restore CM loss after MI (3, 4). Therefore, the current limitations in treating MIs and the need for new therapeutics to stimulate CM proliferation, in a controlled manner, represent an opportunity to improve cardiac function after injury.

A number of studies demonstrate that enhancing CM proliferation after MI improves heart function (5–7). The genetic ablation or pharmacological inhibition of proteins in the Yes-associated protein (YAP) pathway promotes CM cycling. For example, the genetic ablation of the Salvador scaffold protein or MST1 and MST2, two serine/threonine kinases that regulate YAP, promotes robust CM proliferation (8, 9). Alternatively, the overexpression of cyclin-dependent kinase 1 (CDK1), CDK4, cyclin B1, and cyclin D1 or CDK4 and cyclin D1 in combination with Wee1 and TGF-β inhibition promotes CM proliferation in vivo (10). We previously identified that genetic ablation or inhibition of dual-specificity tyrosine phosphorylation–regulated kinase 1A (DYRK1A) induced CM cell cycle entry and improved heart function after ischemia/reperfusion (I/R) MI (11). DYRK1A is an upstream mediator of the Myb-MuvB/dimerization partner, RB-like, E2F, and multi-vulval class B (DREAM) complex, which maintains cell cycle quiescence through suppression of G_1_/S and G_2_/M checkpoints (12). DYRK1A promotes DREAM complex assembly through phosphorylation of LIN52 on residue serine 28 (12). DYRK1A also phosphorylates cyclin D2, targeting it for proteasomal degradation in CMs (13). In contrast, the overexpression of DYRK1A decreased CM proliferation during development in a mouse model of Down syndrome (14). This provides evidence that DYRK1A regulates cell cycle quiescence, and its inhibition can promote reentry of CMs into the cell cycle. However, the mechanisms through which DYRK1A influences cell cycling remain enigmatic.

Our prior experiments have utilized harmine, an inhibitor of DYRK1A that also inhibits monoamine oxidase (11). While harmine has proven effective in promoting CM cycling, it is not without its drawbacks. The drug’s adverse effects, including nausea, vomiting, drowsiness, and impaired concentration, limit its potential clinical use (15). Therefore, our current efforts are focused on developing more specific, orally bioavailable DYRK1A inhibitors that can effectively promote CM cycling without these adverse effects, thereby paving the way for their potential clinical application.

Here, we used computational network modeling to predict mechanisms by which DYRK1A regulates CM cell cycling. These predictions were validated in neonatal rat cardiomyocytes (NRCMs) using selective DYRK1A inhibitors, followed by RNA sequencing. Based on these findings, we tested the DYRK1A inhibitor Leucettinib-92 (LCTB-92) in a mouse model of I/R MI, where it improved heart function and increased CM cycling.

## Results

### Network model predicts mechanisms of DYRK1A-mediated cell cycle activity.

While we have previously found that both genetic and pharmacological inhibition of DYRK1A induces CM cycling (11), the mechanisms by which they do so have not been explored. This led us to develop a network model by manual curation of literature that describes DYRK1A’s role in regulating cell cycling molecules (Supplemental Table 1; supplemental material available online with this article; https://doi.org/10.1172/jci.insight.200429DS1). DYRK1A’s role in cell cycling is believed to be highly conserved across different tissue types, so we broadened our literature review beyond papers that focused on CMs to leverage mechanistic knowledge from other cell types (12, 16–21). We then used these relationships to create a logic-based network model using Netflux (22, 23), where we could mechanistically predict how DYRK1A knockdown increases cell cycle activity (Supplemental Table 2).

The model predicts that in quiescent CMs (baseline), high DYRK1A expression induces DREAM complex formation and RB-1 activity through the direct and indirect degradation of cyclins D and E, respectively (Figure 1A). Upon simulated DYRK1A knockdown, the model predicts an increase in cell cycle entry (DNA replication) through the increase of cyclins D and E, which promotes DREAM complex breakdown and inactivation of RB-1 (Figure 1A). We then validated our model’s accuracy by comparing predictions with experimental results found in literature not used to build the model (Figure 1, B and C, and Supplemental Table 3). Overall, the model’s predictions were highly accurate across multiple experiments performed in CM (100%) and cancer (85%) literature (11, 13, 24–40). Interestingly, the model’s predicted effect of DYRK1A knockout on DNA replication was consistent with CM literature but not consistent with cancer literature (31, 38). DYRK1A may play an alternative role in the context of some cancers (41), which may explain this discrepancy in the literature.

To identify which network nodes are most sensitive to DYRK1A, we simulated a DYRK1A expression concentration response. Node sensitivity was assessed based on predicted EC_50_/IC_50_ values (Figure 1D). Strikingly, nodes further downstream were more sensitive to DYRK1A knockdown, indicating that even partial DYRK1A inhibition may be sufficient to induce cell cycle entry. Finally, we performed a knockdown screen in the context of a 100% DYRK1A knockdown to identify modulators of its response (Figure 1E). We found that knockdown of E2F1, E2F2, and E2F3 transcription factors attenuated the predicted increase in DNA replication upon a DYRK1A knockdown. Additionally, knockdown of cyclin D and E complexes did not attenuate the effect of DYRK1A knockdown on DNA replication, indicating that they may play redundant roles in modulating cell cycle entry. All in all, our robust analysis supports that we have built a reliable model of DYRK1A signaling.

### DYRK1A inhibition promotes cell cycling in NRCMs.

To experimentally validate the network model prediction that DYRK1A inhibition upregulates cyclin D and promotes cell cycle entry, we tested recently reported DYRK1A inhibitors, LCTB-21 and LCTB-92, in NRCMs (42, 43). LCTB-92 and LCTB-21 work by binding to the active site of DYRK1A and preventing kinase activity. Inactive forms of both compounds (ISO-92 and ISO-21, respectively) were used as negative controls along with DMSO. High-content imaging and segmentation of α-actinin, Ki67, cyclin D1, and DAPI (Figure 2A) were used to analyze CM cell cycle activity at single-cell resolution. The network model predicted that DYRK1A inhibition should increase DNA replication in a dose-dependent manner (Figure 2B). Consistent with these predictions, treatment with higher concentrations of LCTB-21 (1 μM) and LCTB-92 (0.1 and 1 μM) increased cell cycle activity as evidenced by a higher percentage of Ki67-positive CMs compared with DMSO and isomeric controls (Figure 2C). The model also predicted a dose-dependent increase in cyclin D levels following DYRK1A inhibition (Figure 2D). Supporting the model predictions, validation experiments showed an increase in cyclin D1 expression in CMs treated with LCTB-21 (1 μM) and LCTB-92 (0.1 and 1 μM) compared with DMSO and isomeric controls (Figure 2E).

Given the substantial increase in both Ki67 and cyclin D1 expression in CMs treated with 1 μM LCTB-92, we further investigated the relationship between these markers and the cell cycle phase under this condition. Analysis of the mean Ki67 and cyclin D1 intensities revealed a shift from a unimodal distribution in cells treated with ISO-92 to a bimodal distribution in LCTB-92–treated CMs (Figure 3A). The shift suggests a discrete bifurcation in Ki67 and cyclin D1 expression that creates distinct subpopulations of CMs (44). Analysis of DNA content and Ki67 (Figure 3B) revealed an increased proportion of CMs in the G_2_/M phase (Figure 3C) and a decreased proportion in the G_0_/4c (Figure 3D) and G_0_/2c phases (“c” refers to DNA content, with 2c and 4c representing diploid and tetraploid levels, respectively) following LCTB-92 treatment compared with ISO-92. This shift toward late cell cycle progression, along with a reduction in multinucleation (Figure 3E), suggests that LCTB-92 promotes true cell division rather than incomplete cell cycle progression.

To uncover the mechanistic differences that explain the efficacy of LCTB-92 versus LCTB-21 in promoting CM proliferation, we performed RNA sequencing on drug-treated NRCMs. Principal component analysis captured 88.6% of the variance across samples and revealed distinct clustering of our LCTB-92 and -21–treated groups (Figure 4A). Differential gene expression analysis was performed in both drug-treated groups with respect to their inactive isomers (Figure 4, B–E). Gene Ontology (GO) enrichment analysis revealed that LCTB-92 treatment was associated with multiple terms related to cell cycle activity, with “regulation of mitotic cell cycle” having the highest gene count (adjusted *P* < 0.05) (Figure 4B). We found a much more pronounced effect in LCTB-92–treated cells, with LCTB-92 having 1,828 unique differentially expressed genes (DEGs), which could explain the more robust effect on cell cycling (Figure 4E). We then visualized the expression levels of genes involved in regulation of mitotic cell cycling and cardiac contraction, identified through our GO term enrichment analysis, across all treatment groups relative to DMSO (Figure 5, A and B). Notably, LCTB-92 treatment elicited a more pronounced transcriptional response, with increased expression of positive regulators and decreased expression of negative regulators of cell cycling. Additionally, we observed a greater decrease in cardiac contraction gene expression in the LCTB-92–treated samples, suggesting more robust CM dedifferentiation, which is a critical component of CM proliferation (45).

To predict drivers of these changes in gene expression, we performed transcription factor enrichment using ChEA3 on the LCTB-92 DEGs (46). We found that E2F1 was a top hit according to mean library rank (Supplemental Table 4). We then used the ChEA3 results to map the network model’s E2F1 node to DEGs associated with cell cycle–related processes (Figure 5C). Several E2F1 targets were also related to cardiac contraction, suggesting it plays a role in CM dedifferentiation.

Despite the enrichment of its downstream targets, the normalized mRNA expression of *E2f1*, *E2f2*, and *E2f3* was not significantly altered in LCTB-92– or LCTB-21–treated cells compared with their respective isomer controls (Supplemental Figure 1, A–D). In contrast, analysis of ChEA3-predicted E2F1/2/3 target genes associated with mitotic processes revealed significant differential expression in both treatment groups in comparison with their respective isomer controls (Supplemental Figure 1E). Given that E2F activity is reported to be modulated at the protein level, our findings suggest that E2F transcription activity is modulated posttranslationally in response to drug treatment (18). Taken together, the integration of model predictions and transcriptomic data suggests that LCTB-92 induces a more defined effect on cell cycling genes compared with LCTB-21, possibly through higher activation of E2F1.

### LCTB-92 promotes CM cell cycle activity and improves heart function after MI.

Based on the in vitro experimental results, we investigated the effects of LCTB-92 in an in vivo model of ischemia/reperfusion myocardial infarction (I/R MI). Specifically, we used our previously described *αDKRC:RLTG* transgenic mouse that restricts Cre recombinase expression to adult CMs that reenter the cell cycle (47). The reporter mouse relies on the activation of the Ki67 promoter, providing an in vivo validation of the Ki67 results in our NRCM experiments. CMs that reenter the cell cycle, based on the activation of a Ki67 promoter, express GFP. *αDKRC:RLTG* mice underwent I/R MI surgeries and were treated with 10 mg/kg LCTB-92 or ISO-92 via daily oral gavage for 10 days (Figure 6A). Serial echocardiography revealed that mice treated with LCTB-92 had significantly improved left ventricular ejection fractions (LVEFs) in comparison with ISO-92 treatment at both 2 and 4 weeks after MI (Figure 6B). However, LCTB-92 treatment did not improve infarct size compared with control 4 weeks after MI, despite improvement in heart function, suggesting that the mechanism of improved cardiac function was not through infarct size reduction (Figure 6, C and D). Additionally, the hearts of mice treated with LCTB-92 had significantly increased number of cycled (GFP^+^) CMs in comparison with ISO-92 treatment (Figure 7, A and B). Interestingly, LCTB-92 did not induce a significant increase of CM cycling in uninjured hearts, suggesting an injury-dependent context for the effects of LCTB-92 on CM cycling.

We then investigated whether the improvement in heart function was linked to the increase in cell cycle activity induced by treatment with LCTB-92 using a previously described mouse model that ablates CMs upon entry into the cell cycle through the Cre-mediated expression of diphtheria toxin (48). *αDKRC:RLTG/DTA* or *+:RLTG/DTA* mice underwent I/R MI and were treated with 10 mg/kg LCTB-92 via daily oral gavage for 10 days (Figure 8A). Serial echocardiography showed that the effects of LCTB-92 were attenuated when cycling CMs were ablated after MI (Figure 8, B–D). Infarct sizes were similar between the 2 groups (Figure 8, E and F). Based on this, we would expect no change in ventricular function in uninjured mice treated with LCTB-92 owing to the absence of a significant increase in cycling CMs. Altogether, the beneficial effects of LCTB-92 on cardiac function after MI require increases in cycling CMs.

### The post-developmental genetic ablation of DYRK1A recapitulates beneficial effects of LCTB-92 treatment.

Previously we observed *aMHC-Cre:DYRK1A^fl/fl^* mice in which CM-specific deletion of DYRK1A starting in development had baseline cardiac hyperplasia and an increase in the expression of cell cycle genes (11). To complement LCTB-92 inhibitor experiments, we investigated the effects of the post-developmental, CM-specific ablation of DYRK1A in adult mice that underwent I/R MI. *cTnnt2-Cre^ERT2^/+:Fucci2aR/Fucci2aR:DYRK1A^fl/fl^* (DYRK1A-KO) or *cTnnt2-Cre^ERT2^/+:Fucci2aR/Fucci2aR* (control) mice were pulsed with tamoxifen for 10 days followed by a 2-week recovery period and then underwent an I/R MI with serial echocardiograms (Figure 9A). DYRK1A-KO mice had improvements in LVEF at 2 and 4 weeks after MI compared with controls (Figure 9, B and C). As with LCTB-92–treated mice that underwent I/R MI, there were no significant differences in infarct size between our DYRK1A-KO and control groups (Figure 9, D and E). We have previously shown that bulk RNA sequencing of CMs isolated from *aMHC-Cre:DYRK1A^fl/fl^* mice 7 days after MI demonstrated upregulation of mitotic and cell cycle–associated genes (11). Although methodological constraints prevented testing whether improvement in ventricular function from post-developmental DYRK1A knockdown is dependent on cell cycling (since our knockout and *αDKRC:DTA* systems relied on Cre recombinase), we can infer that it most likely follows this mechanism based on our LCTB-92 diphtheria toxin subunit A (DTA) experiments. Together, the genetic ablation of DYRK1A in adult CMs recapitulated the effects of LCTB-92 treatment after MI, further supporting the potential therapeutic benefits of DYRK1A inhibition after MI.

## Discussion

Here, we mechanistically propose how small-molecule inhibition of DYRK1A leads to increased cell cycling of CMs both in vitro and in vivo. Our network model accurately predicts DYRK1A-mediated cell cycle activity that we leveraged to inform our in vitro validation experiments in NRCMs. Treatment of NRCMs with LCTB-21 and LCTB-92 exhibited a dose-dependent response on both cyclin D1 and Ki67 expression, validating our model predictions. RNA sequencing of LCTB-21– and LCTB-92–treated NRCMs revealed distinct transcriptional regulation of cell cycling genes, which we mapped to our network model’s predicted node activity via E2F1. LCTB-92 treatment induced CM cycling and improved heart function after I/R MI but did not alter infarct size. However, the beneficial effects of LCTB-92 treatment on heart function were lost when we genetically ablated cycling CMs, indicating that the functional benefits are mediated through cycling CMs. The post-developmental genetic ablation of DYRK1A attenuated the left ventricular dysfunction observed after MI, similarly to the effects of LCTB-92. The two complementary approaches to prevent DYRK1A activity, by pharmacological inhibition or genetic ablation specifically in adult CMs, improved cardiac function after MI, supporting that DYRK1A knockdown after injury can lead to therapeutic benefits.

Our network model was built using literature in various cell types, as DYRK1A’s regulation of cell cycling is believed to be highly conserved. The model predicted experimental outcomes reported in CM literature not used to develop the model, despite the use of predominantly non-CM literature to construct the model. Interestingly, while the model retained high accuracy against experiments from most cancer literature, the model failed to recapitulate experimental results when DYRK1A was knocked down or overexpressed (31, 38). The inconsistency of the model in predicting DYRK1A perturbations in certain cancer cells could be due to the types of cancer cell studied, in which DYRK1A has been reported to be an oncogene. For example, DYRK1A knockdown reduced tumor growth in both in vitro and in vivo models of colon, breast, and neck cancers (49).

We show here that while LCTB-92 promotes cycling of NRCMs and after I/R MI in vivo, it does not induce cycling in uninjured adult hearts. This could be due to the hypoxic environment created after injury. Several reports have demonstrated that hypoxic environments can induce CM proliferation. Puente et al. showed that by exposing NRCMs to hypoxia, they could extend their proliferative window by reducing oxidative stress and DNA damage signaling (50). Similarly, Johnson et al. showed that chronic systemic hypoxia in adult mice induced limited CM proliferation in the right ventricle (51). These findings suggest that the post-injury hypoxic microenvironment may sensitize CMs to DYRK1A inhibition, thereby enhancing the proliferative response observed with LCTB-92 after MI.

The gene dosage of DYRK1A has also been observed to play an important role in CM cycling. Lana-Elola et al. showed that Dp1Tyb mice, a line that has 3 copies of chromosome 21, including DYRK1A, and phenocopies human Down syndrome, have proliferative defects in the embryonic heart (14). Flow cytometry showed an increase in the percentage of CMs in G_1_ phase, indicating a larger quiescent cell population. Additionally, they observed reduced phosphorylated RB-1 and transcription of E2F target genes, consistent with our model predictions. Lastly, RNA sequencing showed that LCTB-21 was able to partially rescue the decreased expression of proliferative pathways in Dp1Tyb embryonic hearts. Treatment with LCTB-92 might be able to induce a more robust recovery of these defects.

The αDKRC mouse model restricts Cre expression to adult CMs that express Ki67. One limitation is that the reporter mouse cannot distinguish between CMs that enter the cell cycle and undergo endoreplication to increase ploidy and CMs that undergo complete mitosis and cytokinesis to promote new daughter cells. Our prior investigations demonstrated that about 10% of adult CMs that enter the cell cycle undergo bona fide proliferation as determined by pairs of clusters of Ki67-expressing CMs (48). With this in mind, we have several hypotheses of how the induction of CM cycling is responsible for the improvement of heart function we see with the treatment of LCTB-92 after I/R. LCTB-92 could be inducing complete CM proliferation that can restore some of the lost myocardium induced by I/R injury and improve the contractile function of the heart. Alternatively, LCTB-92 could also result in the induction of more polyploid CMs, which have been shown to be more responsive to stress through enhanced mitophagy (52). Lastly, cycling CMs may produce paracrine factors that are cardioprotective to the surrounding myocardium. While the use of our *αDKRC:DTA* mice provides evidence that LCTB-92 improves ventricular function in a CM cell cycling–dependent manner, we are unable to truly parse out what aspects of the cycling are beneficial for the myocardium. Additionally, while LCTB-92 administration may improve ventricular function through a combinatorial effect on multiple cell types, our α*DKRC:DTA* mouse experiments demonstrate that DYRK1A inhibition must act at least in part within CMs to confer functional benefit. Further work is necessary to understand the contributing role of LCTB-92–induced cycling CMs in recovery following I/R MI.

The use of our *αDKRC:DTA* mouse to show that LCTB-92’s effect on ventricular function is dependent on CM cell cycling highlights the sensitivity of this subpopulation of CMs. Pride et al. demonstrated in a retrospective analysis of MI patients that there was no significant change in left ventricular function in individuals with infarct sizes less than 15% (53). These findings suggest that a substantial loss of CMs can occur without a measurable decline in ejection fraction. In our study, a marginal reduction of just 0.14% of the total CM population attenuated the benefit of LCTB-92 treatment, highlighting the functional importance and sensitivity of cycling CMs. Cycling CMs, as identified in the αDKRC system, are enriched for cells that have engaged coordinated cell cycle and stress-response programs in vivo, a state that is rare in the adult heart and strongly associated with injury adaptation. These cells are therefore more likely to participate in repair-associated processes, including cytoskeletal remodeling, paracrine signaling, and maintenance of tissue integrity under stress.

It has also been reported that DYRK1A inhibitors trigger the proliferation of pancreatic B cells both in isolated islet cells and in animal models of type 2 diabetes (T2D) (54–56). In addition to DYRK1A inhibition, DYRK1B inhibition appears to contribute to the beneficial effects of the inhibitors used in the diabetic contexts (55). Several structurally different DYRK1A inhibitors have been used, including harmine (57, 58) and LCTB-92. In vivo treatment of the Goto-Kakisaki T2D rat model leads to proliferation of pancreatic β cells and improvement of glycemic control. Like the injury-dependent effect of adult CM cycling we see here, the in vivo effects of LCTB-92 on β cell proliferation are only observed in T2D pathological conditions and not in healthy controls. In both our animal model and the T2D animal models, it appears that LCTB-92 acts to restore homeostasis under pathological conditions.

Most DYRK1A inhibitors also inhibit DYRK1B, as well as some closely related kinases such as the cdc2-like kinases CLK1 and CLK4 and, in some cases, more distantly related kinases like GSK3 (59). LCTB-92 inhibits DYRK1A (IC_50_ value of 1.2 nM), DYRK1B (1.8 nM), DYRK2 (40.2 nM), DYRK3 (19.3 nM), and DYRK4 (117.4 nM) as well as CLK1 (9.2 nM), CLK2 (0.6 nM), CLK3 (811.6 nM), CLK4 (6.0 nM), and GSK3β (4,158 nM) (60). Inhibition of some of these kinases may thus contribute to the effects of the LCTB-92 on CM cycling and heart remodeling after MI. The contribution of DYRK1B inhibition is rather likely, as shown by Bayram et al. (61) and Zhuang et al. (62). Furthermore, CLKs are known to regulate alternative splicing, the modulation of which may affect CM cycling (63). The effects of genetic knockdown of DYRK1A on recovery after MI suggest that the effects of LCTB-92 are mostly mediated through pharmacological inhibition of DYRK1A. Yet inhibition of a few other kinases may complement DYRK1A inhibition in triggering the observed beneficial effects seen following MI.

The potential of the LCTB class of DYRK1A inhibitors to treat acute MI in patients is very attractive. Currently, LCTB-21 is being investigated in a first-in-human phase I trial in healthy volunteers and subjects with Down syndrome and Alzheimer’s disease (NCT06206824). While we show that both LCTB-21 and LCTB-92 induce cycling in CMs in vitro, LCTB-92 showed stronger cell cycling effects, which led to our investigation of LCTB-92 in vivo. Whether LCTB-21 has effects similar to those of LCTB-92 on cardiac function and CM cycling after MI remains to be seen. Importantly, we did not observe tumorigenic effects of LCTB-92 in our studies. The results of this trial, in conjunction with our results using LCTB-92 in a preclinical animal model of MI, are encouraging in that the inhibition of DYRK1A may represent a new first-in-class therapy to treat heart disease. Follow-up studies that recapitulate the results here in more human-relevant model systems would be necessary to investigate the potential of these compounds in treating MI.

## Methods

### Sex as a biological variable.

For in vitro NRCM experiments, cells were isolated from both male and female Sprague-Dawley pups. For in vivo I/R studies, experiments were performed on both male and female mice. We found no statistically significant evidence for sex differences in any of our in vivo experiments.

### Study design.

The aim of this study was to evaluate whether a specific oral inhibitor of DYRK1A would improve left ventricular function after MI and increase adult CM cycling. The study used cultured neonatal rat cardiomyocytes (NRCMs) to examine Ki67 expression in response to LCTB-92, LCTB-21, and their inactive isomers. RNA sequencing was used to investigate changes in the gene expression of NRCMs in response to LCTB-92, LCTB-21, and their inactive isomers. For in vivo studies, we used males and females to investigate sex as a biological variable. We used *αDKRC:RLTG* mice that restrict Cre expression to adult mouse CMs that reenter the cell cycle. *αDKRC:DTA* mice were used to express diphtheria toxin and ablate adult CMs that reenter the cell cycle. *cTnnt2-Cre^ERT2^:DYRK1A^fl/fl^* mice were used to investigate the genetic ablation of DYRK1A in adult CMs. Ischemia/reperfusion (I/R) MIs were performed by ligation of the proximal left anterior descending coronary artery for 60 minutes, followed by restoration for blood flow. This model recapitulated acute MI and reperfusion in humans. Cycling CMs were quantified by counting of EGFP^+^ cells in 10 μm short-axis histological heart sections from *αDKRC:RLTG* mice. Infarct sizes were quantified by examination of serial sections stained with Masson’s trichrome. Sample sizes were selected based on previous experience with similar methods. Mice were randomized to treatment groups based on genotypes for each experiment. Investigators were blinded to genotype when performing surgeries, echocardiography, quantification of EGFP^+^ CMs, and analyses of infarct sizes. No samples were excluded from the study. Details regarding sample sizes, biological replicates, and statistical tests are given in the figure legends and in *Statistics* below.

### Network model construction.

A logic-based differential ordinary equations network model was used to predict mechanisms of DYRK1A-mediated cell cycle activity. The network model was built using Netflux (23), a software that auto-generates a logic-based differential equation model based on a list of reaction rules and parameters (22). This includes assigning the interactions (inhibitory or stimulatory) between molecular components of interest (nodes) as well as the logic-gating strategy (“OR” or “AND” gates). Further information on how this modeling approach works can be found in Kraeutler et al. (22) and Clark et al. (23). A series of 6 primary and review literature articles (Supplemental Table 1) were used to infer the interactions and logic-gating strategy within the model (Supplemental Table 2). Literature was chosen based on its relevance to DYRK1A-mediated cell cycle control and dimerization partner, RB-like, E2F, and multi-vulval class B (DREAM) complex regulation. While literature that used CMs or other relevant experimental models was preferred, we used literature across multiple experimental models (noted in Supplemental Table 1), as this regulatory process is believed to be highly conserved across tissue types. Literature search key terms included proliferation, cell cycling, DREAM complex, and DYRK1A.

Default reaction parameters included reaction weight (w = 1), Hill coefficient (*n* = 1.4), and half-maximal effective concentration (EC_50_ = 0.50), and default node parameters included initial activation (Y_init_ = 0), maximal activation (Y_max_ = 1), and time constant (τ = 1). Parameter optimization was run on input nodes to allow the model to experience change with perturbations at baseline. DYRK1A and B-Myb were then set to a Y_max_ = 0.9 and 0.1, respectively, to establish baseline activity. As in past logic-based differential equation network models (22, 64, 65), species (nodes) refer to a small molecule, gene, protein, or process. Reactions (or edges) are activating and inhibiting relationships between network species (22, 64, 65).

### Network model validation.

Model simulations were compared with experimental data from primary literature that was separate from the literature that was used to build the model’s interactions and logic-gating strategy. Guided by the experimental design of each validation study, we simulated knockdowns or overexpression of specific nodes to see how these perturbations could affect the DNA replication node within the model. Knockdowns were performed by setting of the Y_max_ of a desired node to 0. Overexpression was performed by setting of Y_max_ = 10 for input nodes or by setting of Y_init_ = 10 and τ = 10^9^ for intermediate nodes. All perturbations were performed after the model reached a steady state (<0.05% change in activity for all nodes). Model accuracy was determined by whether simulation results qualitatively matched the statistics from the corresponding experiment in the primary literature (*n* = 19). Validation was performed across cancer and CMs to look at deviations in model predictions across different biological contexts. Experimental figures were replotted with WebPlotDigitizer (66).

### Network model simulations.

We simulated a concentration response by knocking down DYRK1A in increments of 1%. We then looked at the change in activity of the responding nodes to characterize their sensitivity to a DYRK1A knockdown. EC_50_ and IC_50_ of affected nodes were determined by the percentage DYRK1A knockdown at which their normalized activity was changed by 50%. A knockdown screen was conducted through simulated 100% knockdowns on each node of the model at baseline activity, where DYRK1A is highly active (Y_max_ = 1). We then examined the change in activity of the network in response to each individual knocked-down node. We simulated “low,” “medium,” and “high” DYRK1A inhibitor concentrations by knocking down DYRK1A in increments of 15%. Our concentrations corresponded to these simulated knockdown values: low, 15%; medium, 30%; and high, 45%. The results were then compared with in vitro data of NRCMs that received the LCTB-92 and LCTB-21 DYRK1A inhibitors.

### NRCM isolation, immunostaining, and image analysis.

CMs were isolated from 1- to 2-day-old Sprague-Dawley rats using a Neomyt isolation kit (Cellutron). Each cell isolation was a mixture of CMs from all 9–12 male and female pups from that litter. The cells were cultured in plating medium (low-glucose Dulbecco’s modified Eagle medium [DMEM], 17% M199, 10% horse serum, 5% fetal bovine serum, 1% l-glutamine, 10 U/mL penicillin, and 50 mg/mL streptomycin) at a density of 30,000 cells per well in a 96-well Corning CellBIND plate coated with SureCoat (Cellutron). After being cultured for 48 hours, the cells were serum starved in WE+B medium (William’s E medium without phenol red [Gibco] supplemented with Primary Hepatocyte Cell Maintenance Cocktail B [Gibco] containing Penicillin-Streptomycin, ITS+ [insulin, transferrin, selenium complex, BSA, and linoleic acid], GlutaMAX, and HEPES [all from Gibco]) for 4 hours before drug treatment. CMs were then treated with the DYRK1A inhibitor LCTB-92 or LCTB-21 (0.01–1 μM) or respective isomeric and DMSO (0.01%) controls in serum-free medium for 48 hours.

Cells were then fixed in 4% paraformaldehyde and fluorescently labeled with anti–α-actinin (MilliporeSigma) or cardiac troponin T (Abcam), anti-Ki67 (Invitrogen), anti–cyclin D1 (Abcam), and DAPI (Life Technologies). The specific primary antibodies used are listed in Table 1. Stained cells were imaged with the Operetta CLS high-content imaging system (PerkinElmer) using a ×10, 0.3 NA objective. Image analysis was performed using a CellProfiler pipeline for cell segmentation to quantify the percentage of CMs positive for Ki67 or cyclin D1 (67). Thresholds for mean nuclear Ki67 or cyclin D1 intensity were set manually to approximate the local minimum of the bimodal distribution. Multinucleated CMs were identified by measurement of the distance between adjacent nuclei, with a threshold of less than 2 pixels. Cell cycle phase was assessed by DNA content analysis combined with Ki67 positivity as described in previous studies (68, 69).

### Gene expression analysis.

NRCMs were isolated and cultured as previously described, then seeded at a density of 1,000,000 cells per well in 6-well Corning CellBIND plates coated with SureCoat (Cellutron). Cells were treated with 1 μM of either LCTB-92 or LCTB-21, their respective isomeric controls, or DMSO (*n* = 3 per condition), following the same treatment protocol as described above. RNA extraction, cDNA library preparation (including adapter ligation), and sequencing were performed by Genewiz Inc. using an Illumina HiSeq system. FASTQ files were processed using a Nextflow pipeline (STAR + Salmon), in which reads were aligned with STAR and transcript abundance was quantified using Salmon (70). Principal component analysis and differential gene expression analysis were conducted using the DESeq2 package in R (71). Gene Ontology (GO) enrichment analysis was performed with the ClusterProfiler package in R (72). Transcription factor enrichment was assessed using the ChEA3 API platform, with transcription factors ranked by average rank across all libraries (46). To integrate RNA sequencing data with our network model, we linked the E2F1 node to its target genes identified via ChEA3 and mapped these to gene sets associated with DNA replication, cytokinesis, and cardiac contraction GO terms.

### Mice.

*αDKRC* (*αMHC-MerDreMer-Ki67p-RoxedCre:Rox-Lox-tdTomato-eGFP*) mice were generated and maintained as previously described. *C57BL/6J* (JAX stock 000664), *RC:RLTG* [*B6.Cg-Gt(ROSA)26Sor^tm1.2(CAG-tdTomato,-EGFP)Pjen^/J*], and *ROSA-DTA* [*B6.129P2-Gt(ROSA)26Sor^tm1(DTA)Lky^/J*] mice were purchased from The Jackson Laboratory. *CAG-STOP-Fucci2aR* mice were obtained from the European Mouse Mutant Archive. *cTnnt2-Cre^ERT2^* mice were provided by Chenlen Cai (Indiana University School of Medicine, Indianapolis, Indiana USA). The *αDKRC:RLTG*, *αDKRC:DTA*, *cTnnt2-Cre^ERT2^:Fucci2aR*, and *cTnnt2-Cre^ERT2^:Fucci2aR:DYRK1A^fl/fl^* mice were created by standard breeding methods, and all mice were genotyped. DYRK1A knockdown was confirmed by qPCR as described in our previous article (11). We were unable to show protein-level knockdown of DYRK1A owing to the known limitations of DYRK1A antibody performance in tissue lysates.

### Compounds.

LCTB-92, ISO-92, LCTB-21, and ISO-21 were provided by Perha Pharmaceuticals. For experiments using NRCMs, LCTB-92, ISO-92, LCTB-21, and ISO-21 were dissolved in DMSO and added to cell media to give a final concentration of 0.01 μM, 0.1 μM, or 1 μM. For in vivo experiments, LCTB-92 or ISO-92 was dissolved in 0.5% carboxymethylcellulose in sterile water, and 1 mg/kg was given daily by oral gavage.

### Left anterior descending artery I/R MI surgery.

All animal care and surgeries were in accordance with the University of Virginia (UVA) Animal Care and Use Committee (ACUC) Policy on Rodent Surgery and Perioperative Care under an ACUC-approved animal protocol (UVA ACUC Wolf Lab protocol 4080). The individual performing surgeries was blinded to the mouse genotypes and treatments. Male and female 10- to 12-week-old mice were randomized to MI surgeries, weighed, and then anesthetized in an induction chamber using the gas anesthetic isoflurane (3% volume/weight, oxygen 500 mL/min). The animal was placed in the supine position on a face mask connected to the anesthesia system. Isoflurane was adjusted to provide a maintenance level (1.8%–2.2% volume/weight, oxygen 500 mL/min) throughout the procedure. Anesthesia was monitored closely by the following methods: (a) depth and rate of respiration; (b) heart rate by ECG; (c) mucous membrane color; (d) body temperature via PhysioSuite Homoeothermic Temperature Monitoring System (Kent Scientific); (e) reflexes and toe pinch; and (f) overall appearance of muscle relaxation. All surgical procedures were carried out with a stereo microscope exclusively for small-animal surgery. Normal body temperature was maintained using an electrical heating pad on a feedback system via rectal probe.

To prepare for surgery, the mouse was first given subcutaneous fluids (veterinary Normosol; Pfizer), and atropine as a pre-anesthetic to decrease mucus secretion and to prevent gag reflex during intubation. The neck and chest wall were shaved, and then prepped 3 times with alternating wipes of povidone-iodine and 70% ethanol. Mice were placed in the supine position on a heating pad, and an endotracheal intubation was performed under direct laryngoscopy with a lighted fiber optic stylus to visualize the vocal cords and insert the tracheal tube. The endotracheal tube was then connected to the automated ventilator (Kent Scientific Inc.), and the mouse was ventilated (tidal volume = 1.0 mL, rate = 120 breaths/min). Bupivacaine was infiltrated subcutaneously into the area of the left third and fourth intercostal spaces. A left anterior thoracotomy was performed using sterile technique by making a small subcutaneous incision lateral from the sternum with sharp scissors. The pectoralis muscle groups were identified and separated by blunt dissection, and held open using fixed retractors. The thoracic cage was exposed, and the third intercostal space was identified. Incision into the third intercostal space was done by a combination of blunt micro-scissors and micro-cauterizing tool in order to minimize bleeding. Retractors were then repositioned onto the upper and lower ribs in order to visualize the heart. The pericardium was then bluntly dissected in order to expose the anterior view of the heart. The left anterior descending artery (LAD) on the surface of the left ventricular wall was identified, an 8-0 prolene suture was placed through the myocardium into the anterolateral left ventricular wall underneath the LAD at the level of the lower atrium (1 mm below the left auricle), and the suture was tied. Occlusion of blood flow was observed by blanching of the heart muscle, and ST elevation was confirmed by ECG. After 60 minutes, the ligature was removed. The chest wall, facial planes, and skin were sutured, and the mouse was allowed to recover and treated with analgesics. In sham control mice, the entire procedure was identical except for the ligation of the LAD. The mortality rate associated with surgeries was approximately 10%.

### Histology and immunohistochemistry.

Hearts were excised and fixed in 10% neutral-buffered formalin (Fisher Inc.) for a minimum of 4 hours before embedding in paraffin. Ten-micrometer sections were prepared in short-axis orientation by microtome with 8 sections per glass slide. Paraffin was removed, and the tissue sections were rehydrated using xylene and serial ethanol wash steps. Antigen retrieval was performed by incubation of tissue sections in boiling 1× Unmasking solution (Vector Laboratories H-3300) for 22 minutes. After cooling to room temperature, the tissue sections were treated with Sudan Black to quench autofluorescence. Briefly, tissue sections were incubated in 0.1% Sudan Black (MilliporeSigma, catalog 199664) in 1× PBS and 70% ethanol at room temperature for 20 minutes to quench autofluorescence, followed by three 5-minute washes in 1× PBS containing 0.02% Tween 20, and a final 5-minute wash in 1× PBS.

### Quantification of CM cycling.

Images were obtained using a Leica DM2500 fluorescence microscopy system with a Leica DFC7000 T fluorescence color camera or a Leica THUNDER imaging system and Leica LAS X Multi Channel Acquisition software. For imaging, fluorescence channels were calibrated to background of control sections stained with secondary antibodies alone. EGFP^+^ CMs were visualized using a YFP filter and quantified from six to eight 10-μm short-axis sections of 3 slides per animal separated by about 400 μm per slide. The infarct zone was identified using wheat germ agglutinin (WGA). The non-infarct zone was divided into thirds with the 2 regions adjacent to the infarct defined as the border zones and the middle region defined as the remote zone. EGFP^+^ CMs present in the same location on sequential slides were counted once among all sections to avoid overrepresentation of EGFP^+^ cells.

### Echocardiography.

Mice were anesthetized with isoflurane and gently restrained on a Vevo integrated rail system. The system included a physiological monitoring unit consisting of a heating board with integrated ECG electrodes. The table temperature was maintained at approximately 38°C using a rectal probe for temperature feedback. Hair was gently removed, and ultrasound contact gel (warmed to 37°C) was applied to the chest. Echocardiography was performed using a high-frequency 30 MHz linear transducer and a Vevo 1100 (Visual Sonics) system similarly to previously described methods (47, 48). Initial B-mode images were obtained in the parasternal long axis with the apex and aortic valves visualized. Next, B-mode short-axis images were obtained by turning the ultrasound probe approximately 90° and identifying the mitral valve papillary muscles. The parasternal long-axis images were analyzed using VevoView software to calculate left ventricular dimensions. The individuals performing and analyzing the echocardiography were blinded to the treatment groups.

### Statistics.

GraphPad Prism 9 (GraphPad Software Inc.) was used for statistical analyses. The data were analyzed for normal distribution using Anderson-Darling, D’Agostino and Pearson, Shapiro-Wilk, and Kolmogorov-Smirnov tests in GraphPad. One-way ANOVAs with Tukey’s test corrections for multiple comparisons, 2-way repeated-measures ANOVA with Bonferroni’s test for multiple comparisons, and 2-tailed Student’s *t* tests were used. We used a calculator from the IACUC at Boston University, including an Excel template for calculation based on means/standard deviations and proportions (https://www.bu.edu/research/forms-policies/iacuc-sample-size-calculations/). For experiments quantifying EGFP^+^ CMs, we assumed a type I (α) error of 0.05, a difference (Δ) of 2-fold (~2–3 EGFP^+^ cells in sham heart sections and 4–6 EGFP^+^ cells in MI heart sections), a standard deviation of 2–3, and a power of 0.9. We calculated that 9 mice would be needed in each group. For the echocardiography experiments, we assumed a type I (α) error of 0.05, a difference (Δ) of approximately 10% (ejection fraction ~30% in the MI control group and ~40% in the drug-treated group), a standard deviation of 5%, and a power of 0.95. We calculated that a minimum of about 7 mice would be needed in each group.

### Study approval.

All mice were housed and maintained in accordance with a protocol approved by the University of Virginia (UVA) Animal Care and Use Committee (ACUC) (UVA ACUC Wolf Lab protocol 4080). Rats were housed and maintained in accordance with a protocol approved by the UVA ACUC (UVA ACUC Saucerman Lab protocol 4080).

### Data availability.

Data associated with this study are present in the paper or its supplemental materials. RNA sequencing data were deposited in the Gene Expression Omnibus (GEO) database maintained by the NCBI under accession number GSE341619. Netflux code is available at https://github.com/saucermanlab/Netflux (commit ID 1af412a). Mouse data are available by request.

## Author contributions

AJE performed echocardiography and image analysis of infarct size. JJS and MJW designed the study, obtained funding, supervised the work, and edited the manuscript. BCM performed the network modeling, RNA sequencing, and experiments on tissue sections, and wrote the manuscript. KLW performed the in vitro experiments and image analysis, and wrote the relevant sections of the manuscript. BNH, MW, and CZ performed network modeling. AY, LAB, KS, DH, LM, and MJW designed the mouse breeding and surgeries, and performed and analyzed the echocardiography, histology, and immunohistochemistry. AY performed the mouse surgeries and echocardiograms. LM, MFL, and ED provided the synthesized and purified LCTB-92, ISO-92, LCTB-21, and ISO-21 and expertise regarding the design of the mouse experiments.

## Conflict of interest

LM is a founder of Perha Pharmaceuticals. LM and ED are coinventors on the Leucettinib patents (new imidazolone derivatives as inhibitors of protein kinases, in particular, DYRK1A, CLK1, and/or CLK4; EP4143185, PcT/EP2021/061349, Wo2021219828a1, EP4173675a1, EP4173673, and EP4173674).

## Funding support

This work is the result of NIH funding, in whole or in part, and is subject to the NIH Public Access Policy. Through acceptance of this federal funding, the NIH has been given a right to make the work publicly available in PubMed Central.

- National Institutes of Health grants R01HL158718 to MJW, R01HL162925 to JJS and MJW, R01HL160665 to JJS, and T32GM13661 to BCM.

## Supplementary Material

Supplemental data

Supplemental table 1

Supplemental table 2

Supplemental table 3

Supplemental table 4

Supporting data values

## Figures and Tables

**Figure 1:**
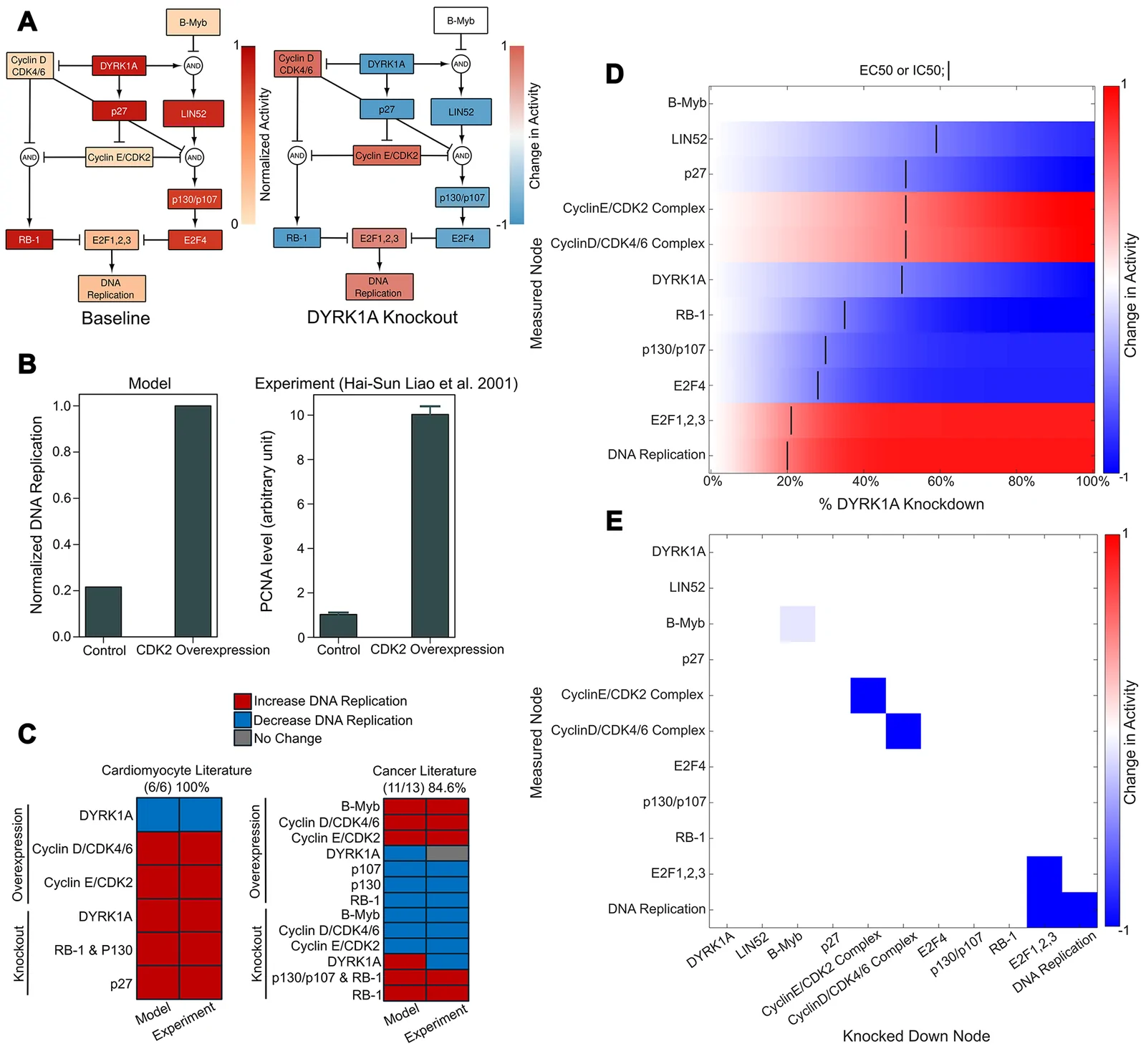
Network model predicts mechanisms of DYRK1A-mediated CM cell cycle activity. (**A**) Model predicts response to active DYRK1A (left) and DYRK1A knockout (right). (**B**) Model simulation of CDK2 overexpression’s effect on DNA replication (left) compared with experimental data found in the literature (right). (**C**) Other simulations were compared with experiments in both CM (*n* = 6) and cancer literature (*n* = 13) (*n* = number of primary literature articles). Validation accuracy was measured by the number of predictions that match literature results/simulations performed. (**D**) Concentration response showing node activity resulting from increasing levels of DYRK1A knockdown. IC_50_ and EC_50_ values indicate the percentage DYRK1A knockdown at which a node increases or decreases by 50% (determined by 50% change in normalized activity). (**E**) Knockdown screen showing network model’s response to each node being 100% knocked down. Nodes were knocked out 1 at a time by setting of their Y_max_ to 0. Simulations were performed under a 100% DYRK1A knockdown.

**Figure 2:**
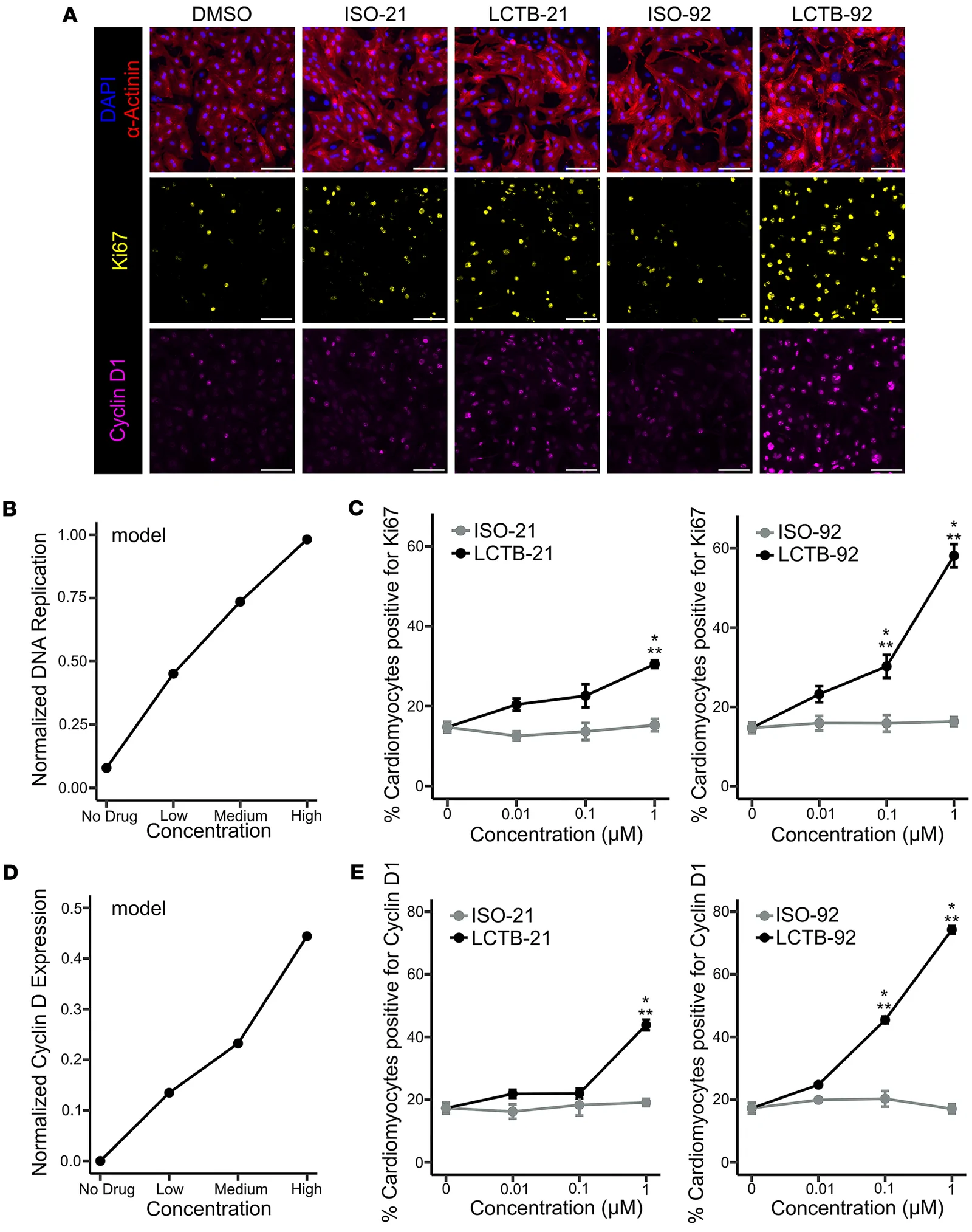
The DYRK1A inhibitor LCTB-92 increases cell cycle activity of NRCMs, validating the network model. (**A**) Representative images of CMs treated with DYRK1A inhibitors (LCTB-21, LCTB-92) or controls (DMSO, ISO-21, ISO-92) and labeled with DAPI (blue), α-actinin (red), Ki67 (yellow), and cyclin D1 (magenta). Scale bars: 100 μm. (**B** and **C**) Network model prediction of DNA replication (**B**) and experimental quantification of the percentage of CMs expressing Ki67 (**C**) in response to DYRK1A inhibition (*N* = 2, *n* = 6). (**D** and **E**) Network model prediction of cyclin D expression (**D**) and experimental quantification of the percentage of CMs expressing cyclin D1 (**E**) in response to DYRK1A inhibition (*N* = 1, *n* = 3). Data are shown as mean ± SEM. Statistical significance for **C** and **E** was determined by 1-way ANOVA followed by Tukey’s multiple-comparison test. *P < 0.05, LCTB treatments compared with the respective isomeric control; **P < 0.05, LCTB treatments compared with DMSO (0 mM) control. *N* represents number of NRCM isolations, and *n* represents technical replicates.

**Figure 3:**
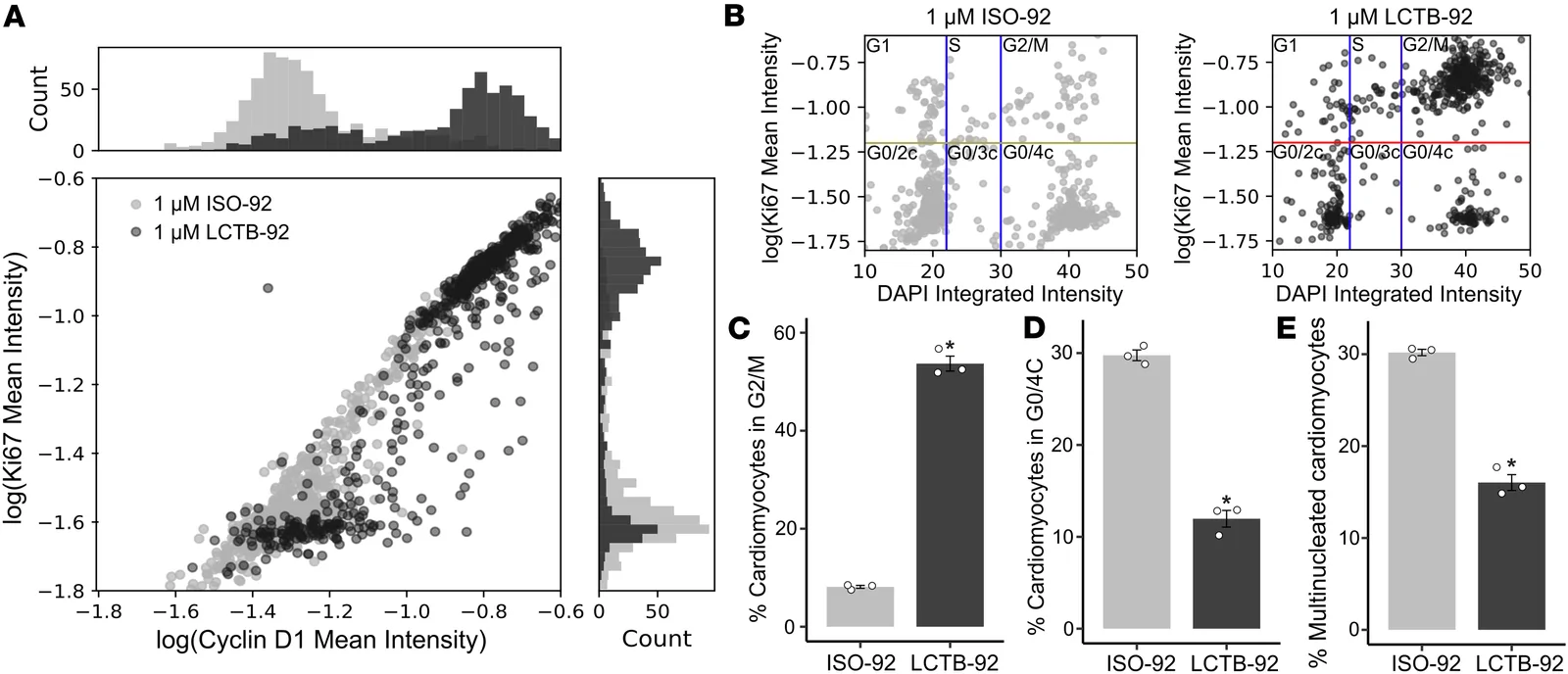
LCTB-92 increases late-stage cell cycle activity and decreases multinucleation in NRCMs. (**A**) Single-cell correlation between Ki67 and cyclin D1 expression levels with histograms illustrating the distribution of intensities for each marker. (**B**) Bivariate analysis of DNA content and Ki67 to separate out cell cycle phase. DNA content thresholds separating 2c, 3c, and 4c nuclei are shown as blue vertical lines. Ki67-positive threshold is shown as a red horizontal line. (**C** and **D**) Percentage of CMs in G_2_/M (**C**) and G_0_/4c (**D**) phases from **B**. (**E**) Percentage of multinucleated CMs (*N* = 2, *n* = 6). Data are shown as mean ± SEM. Statistical significance for **C**–**E** was determined by *t* test. *P < 0.05. *N* represents number of NRCM isolations, and *n* represents technical replicates.

**Figure 4:**
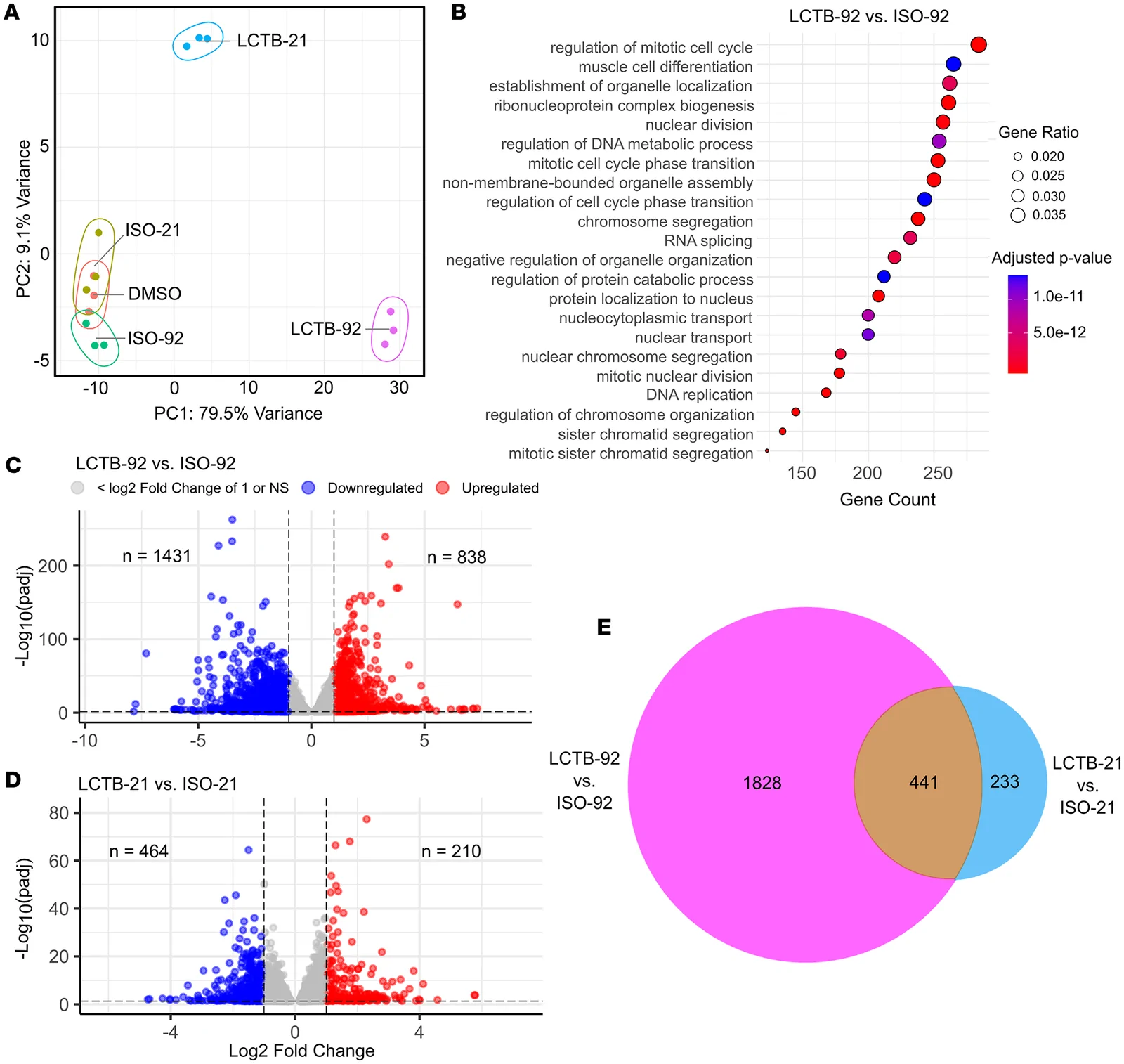
LCTB-92–treated NRCMs exhibit transcriptomic signatures associated with cell division. (**A**) Principal component analysis of drug-treated and respective control NRCM samples. (**B**) Gene Ontology (GO) enrichment analysis of LCTB-92–treated NRCMs with respect to its inactive isomer (ISO-92). (**C**–**E**) Number of significantly differentially expressed genes with at least 2-fold increase in LCTB-92–treated (**C**) or LCTB-21–treated (**D**) NRCMs in comparison with ISO-92 and ISO-21, respectively; and the commonality between both treatment groups (**E**). GO term enrichment analysis was performed with a *P* value cutoff of 0.01 and a *q* value cutoff of 0.05.

**Figure 5:**
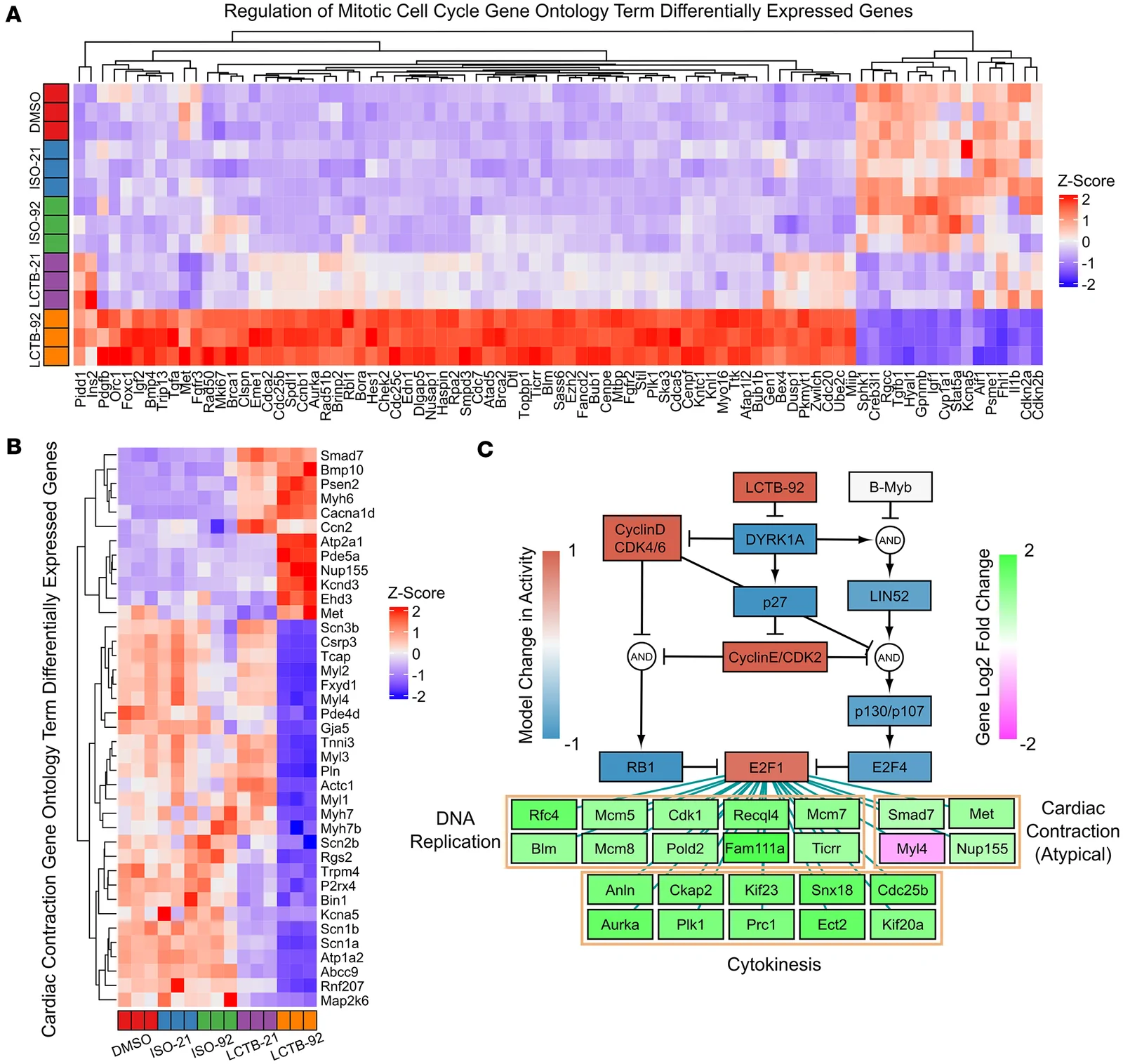
Network model predicts drug-induced E2F1-mediated transcriptional activity. (**A** and **B**) *Z* score of differential gene expression in genes pulled from “regulation of mitotic cell cycle” (**A**) and “cardiac contraction” (**B**) GO terms. (**C**) Network model simulation of LCTB-92 inhibition of DYRK1A and corresponding gene expression of cytokinesis, DNA replication, and cardiac contraction E2F1 target genes. E2F1 target genes were pulled from ChEA3-predicted targets found in Supplemental Table 4.

**Figure 6:**
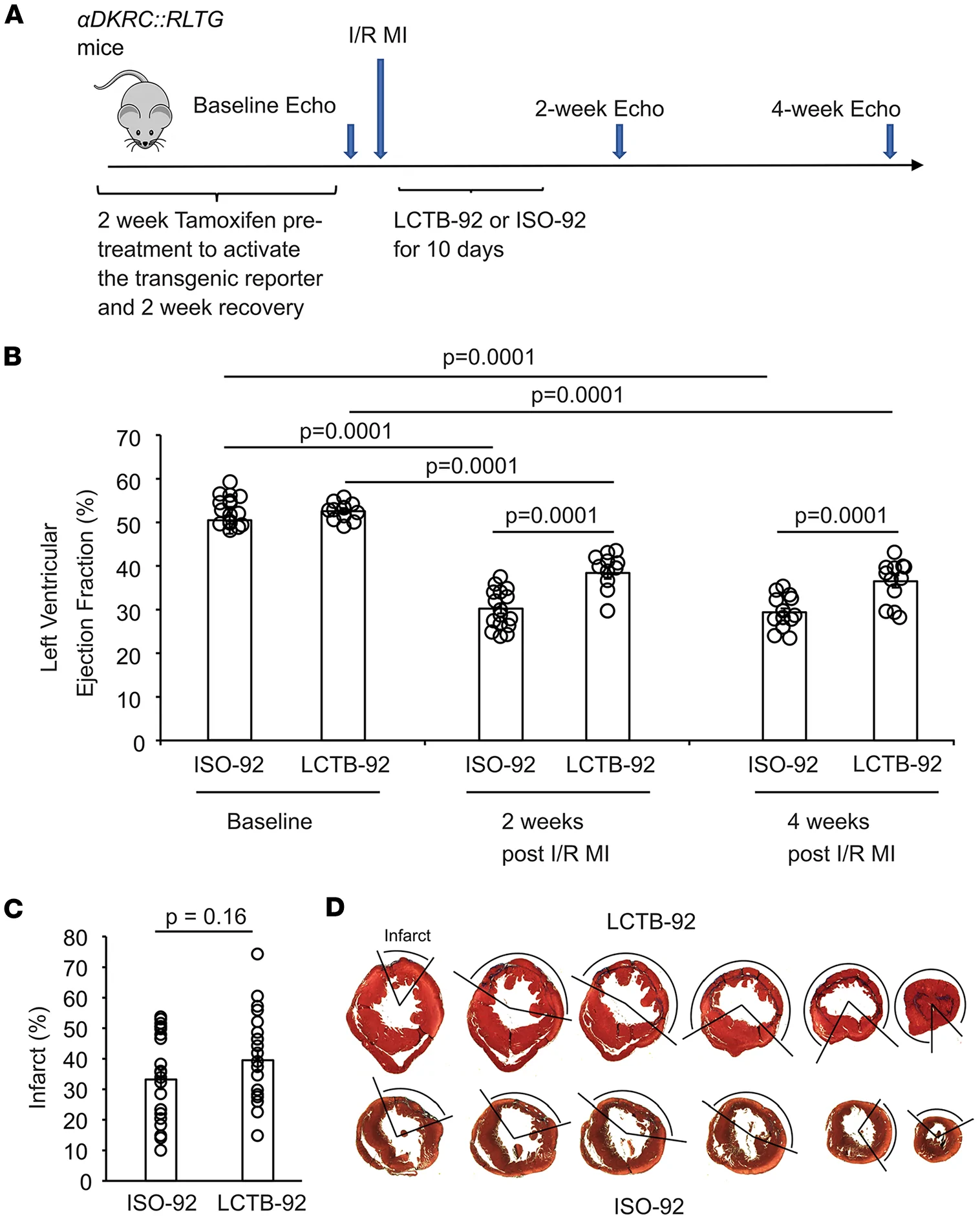
LCTB-92 improves heart function after I/R MI. (**A**) Schematic of 12-week-old *αDKRC:RLTG* mice that were treated with LCTB-92 or isomeric control for 10 days (10 mg/kg) following I/R MI. (**B**) Cardiac function measurements of *αDKRC:RLTG* mice treated with LCTB-92 or isomeric control after I/R MI, showing left ventricular ejection fraction (LVEF) 2 and 4 weeks after MI. Data are shown as mean ± SD, and circles are measurements of individual animals. *N* = 20 for ISO-92 group and *N* = 19 for LCTB-92 group. *P* values were calculated using 2-way ANOVA followed by Bonferroni’s multiple-comparison test. (**C** and **D**) Average infarct size (percent) across whole heart sections from hearts treated with LCTB-92 or isomeric control 4 weeks after MI (**C**) with representative images (**D**). Data are shown as mean ± SD, and circles are measurements of individual animals. *N* = 18 for ISO-92 group and *N* = 17 for LCTB-92 group. Two-sample *t* test was performed for **C**.

**Figure 7:**
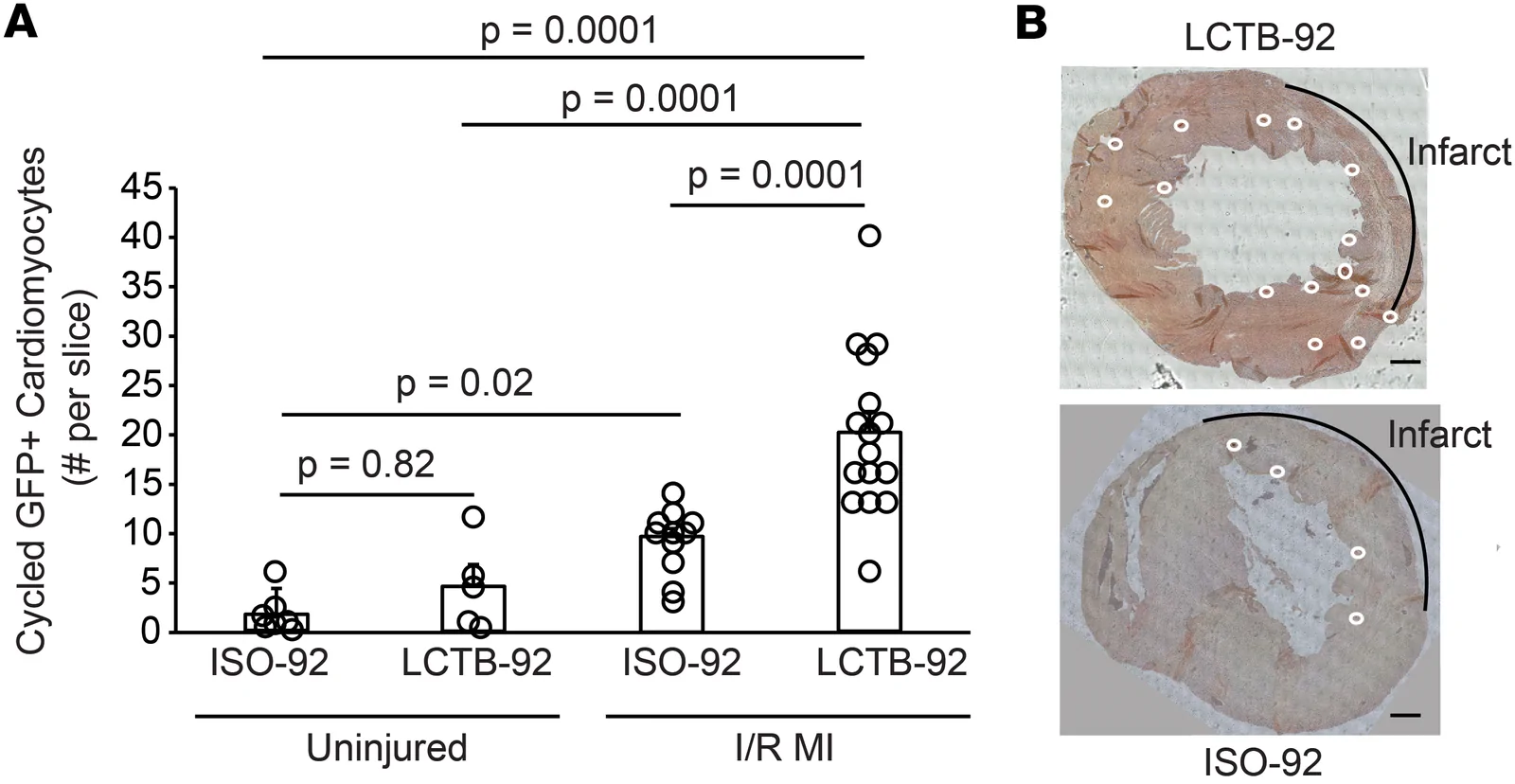
LCTB-92 promotes CM reentry after I/R MI. (**A** and **B**) Quantification of the number of GFP^+^ CMs collected from the midventricular section of hearts treated with LCTB-92 or isomeric control 4 weeks after MI (**A**) with representative images of GFP^+^ CMs (white circles) indicating cell cycle reentry (**B**). *P* values were calculated using 2-way ANOVA followed by Bonferroni’s multiple-comparison test. *N* = 7 animals for ISO-92 uninjured, *N* = 8 animals for LCTB-92 uninjured, *N* = 15 animals for ISO-92 I/R MI, and *N* = 16 animals for LCTB-92 I/R MI.

**Figure 8:**
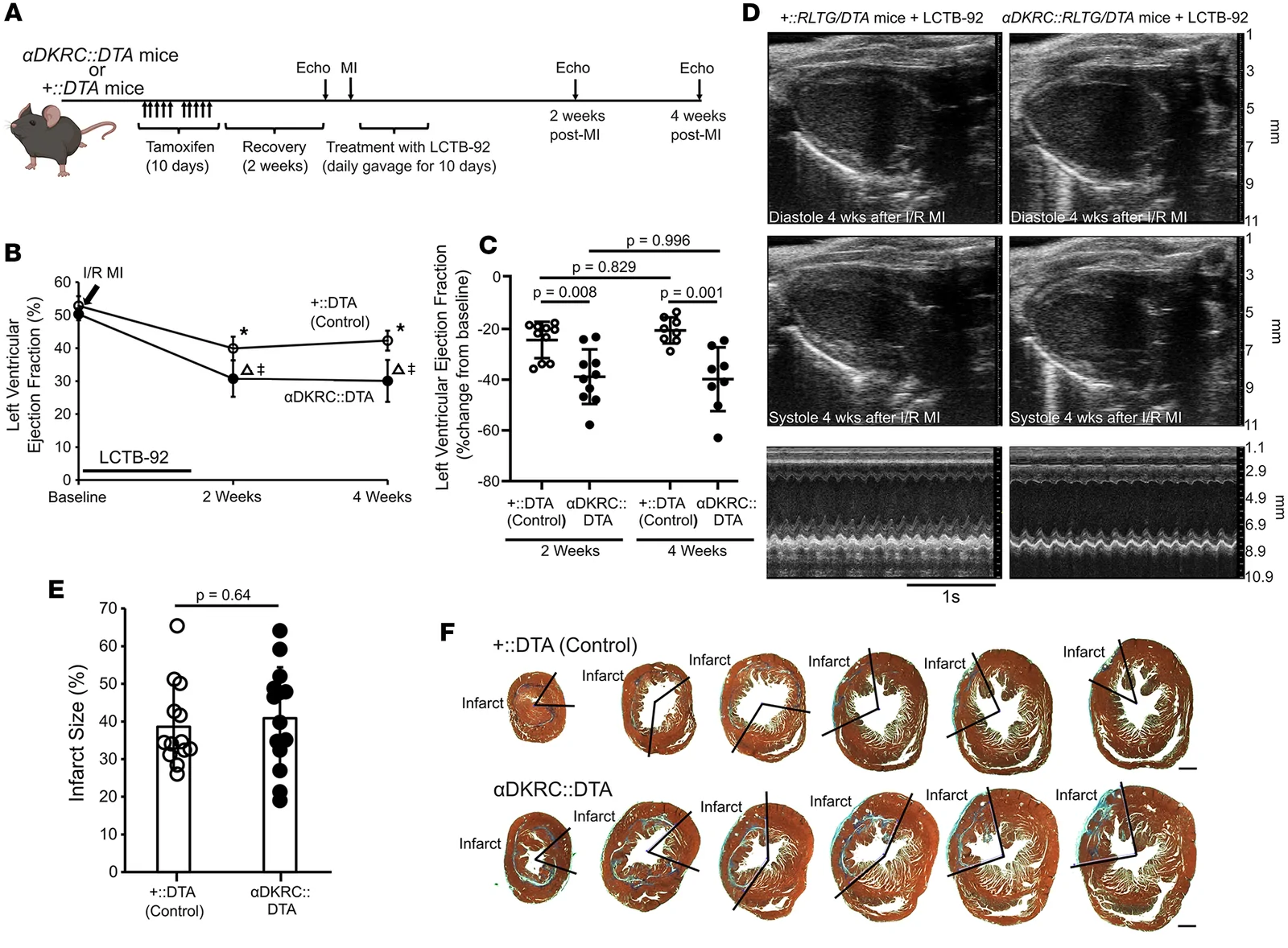
Ablation of cycling CMs attenuates beneficial effects of LCTB-92 treatment. (**A**) Schematic of treatment of 12-week-old *αDKRC:RLTG/DTA* or *+:RLTG/DTA* mice with LCTB-92 (10 mg/kg) for 10 days following I/R MI. (**B** and **C**) Cardiac function measurements of *αDKRC:RLTG/DTA* or control (*+:RLTG/DTA*) mice treated with LCTB-92 or isomeric control after I/R MI, showing absolute (percent) (**B**) and percent change (from baseline) LVEF (**C**) 2 and 4 weeks after MI. *N* = 10 animals per group. Values are mean ± SD. *P* values were calculated using 2-way ANOVA followed by Bonferroni’s multiple-comparison test. *P = 0.005 for each value compared with baseline; ^Δ^*P* = 0.001 for each value compared with baseline; **^ǂ^***P* = 0.05 for *αDKRC:RLTG/DTA* vs. control. (**D**) Representative echocardiography parasternal long-axis views of *αDKRC:RLTG/DTA* or control (*+:RLTG/DTA*) mice treated with LCTB-92 at 4 weeks after I/R MI. (**E** and **F**) Average infarct size (percent) across whole heart sections from *αDKRC:RLTG/DTA* or control (*+:RLTG/DTA*) hearts treated with LCTB-92 at 4 weeks after MI (**E**) with representative images (**F**). Values are mean ± SD. Two-sample *t* test was performed for **E**.

**Figure 9:**
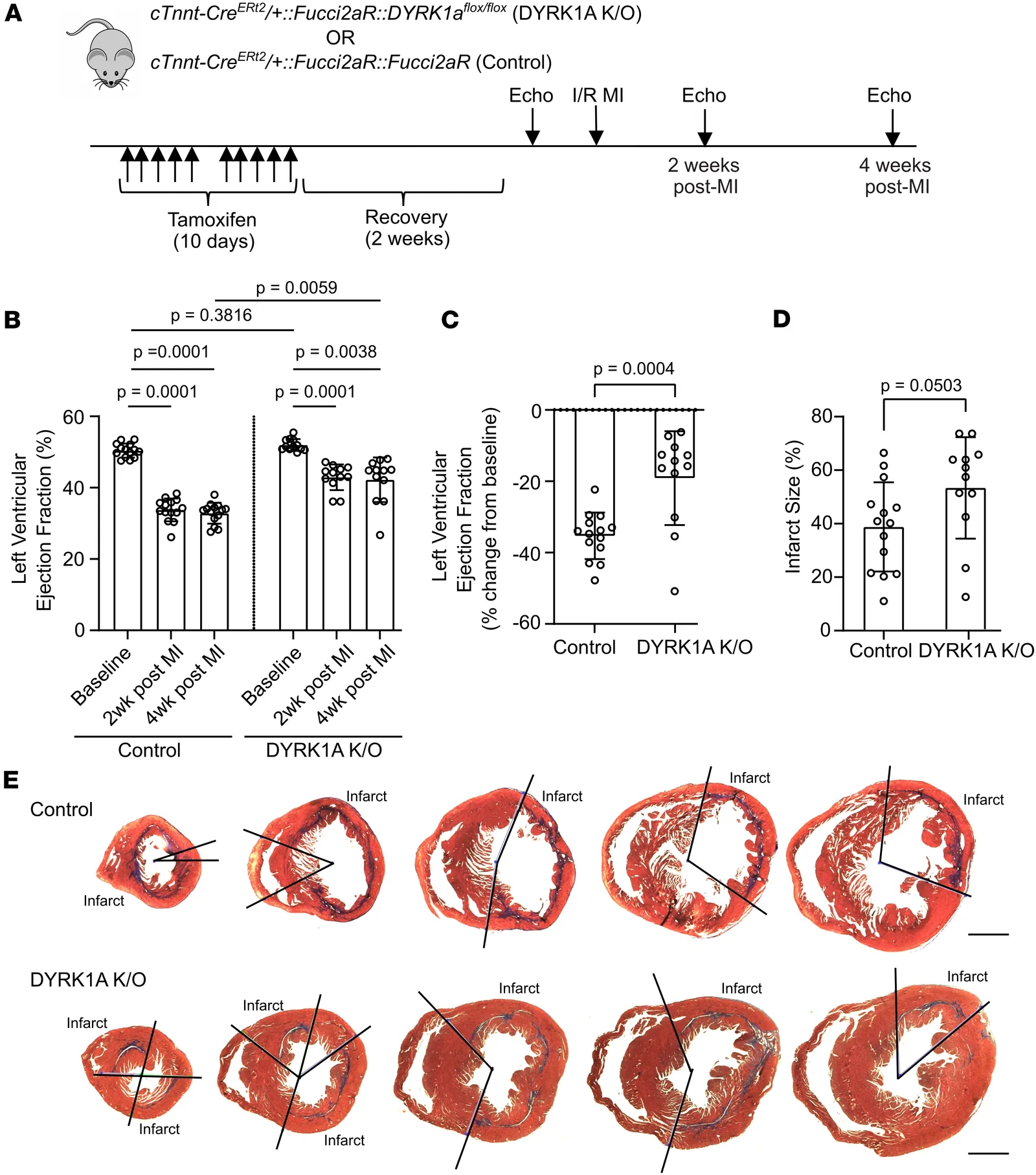
Post-developmental CM-specific deletion of DYRK1A recapitulates improved heart function induced by LCTB-92 treatment after I/R MI. (**A**) Schematic of 12-week-old *cTnnt2-Cre^ERT2^/+:Fucci2aR/Fucci2aR:DYRK1A^fl/fl^* (CM-specific DYRK1A-KO) or *cTnnt2-Cre^ERT2^/+:Fucci2aR/Fucci2aR* (control) mice that underwent I/R MI. (**B** and **C**) Absolute (percent) (**B**) 2 and 4 weeks after MI and percent change (from baseline) LVEF (**C**) 4 weeks after MI. *P* values were calculated using 2-way ANOVA followed by Bonferroni’s multiple-comparison test. *N* = 18 for DYRK1A-KO animals and *N* = 17 for control animals. (**D** and **E**) Average infarct size (percent) across whole heart sections from DYRK1A-KO or control hearts 4 weeks after MI (**D**) with representative images (**E**). *N* = 12 for DYRK1A-KO animals and *N* = 14 for control animals. *P* values were calculated by 2-sample *t* test.

**Table 1:**
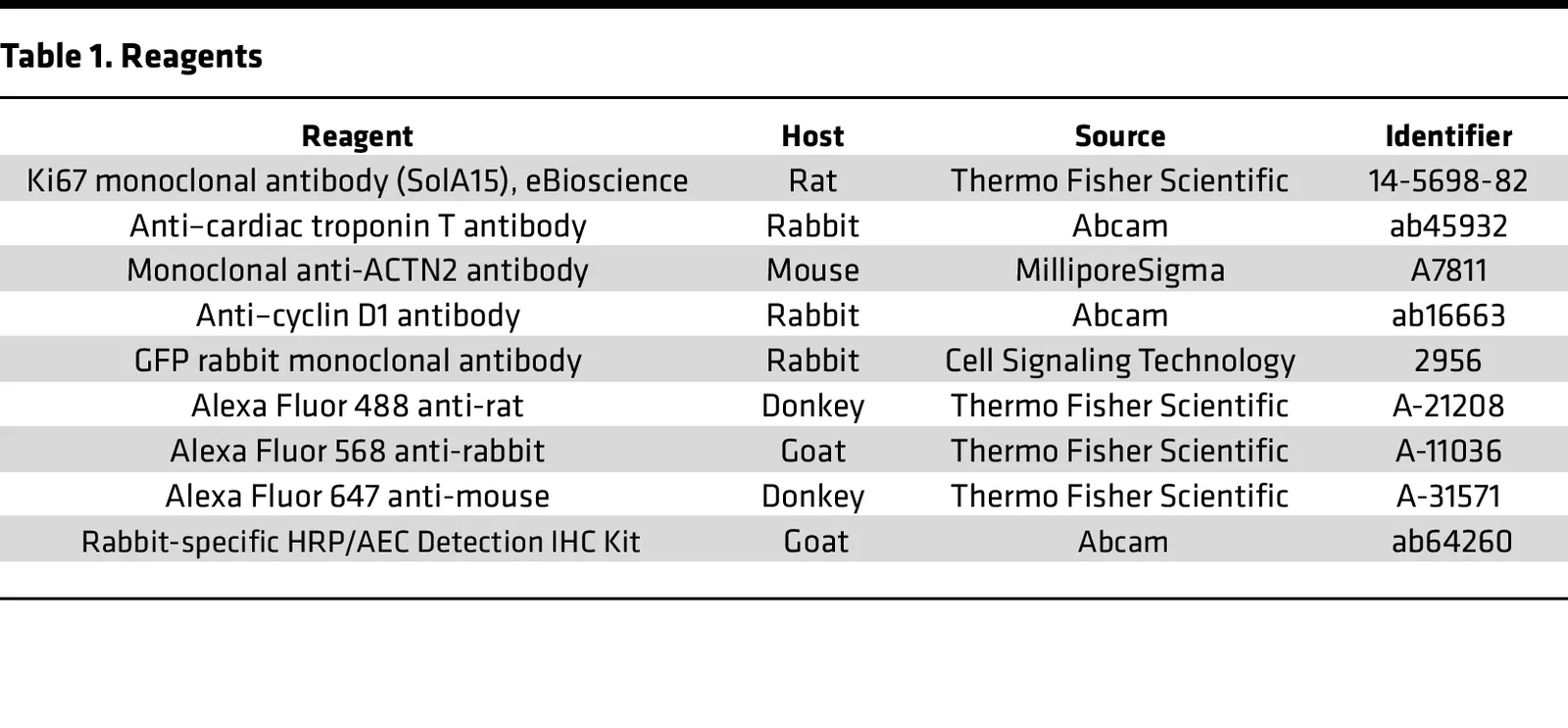
Reagents

## Version 1 09/17/2026

In-Press Preview

## Version 2 10/22/2026

Electronic publication
