# Supplementary material for: Network modeling predicts how DYRK1A inhibition promotes cardiomyocyte cycling after ischemia/reperfusion injury
Source: JCI Insight. 2026 Sep 17;11(20):e200429. doi: 10.1172/jci.insight.200429 (PMC13726558; doi:10.1172/jci.insight.200429)
Supplement: Supplementary file 1 [file jciinsight-11-200429-s140.pdf]

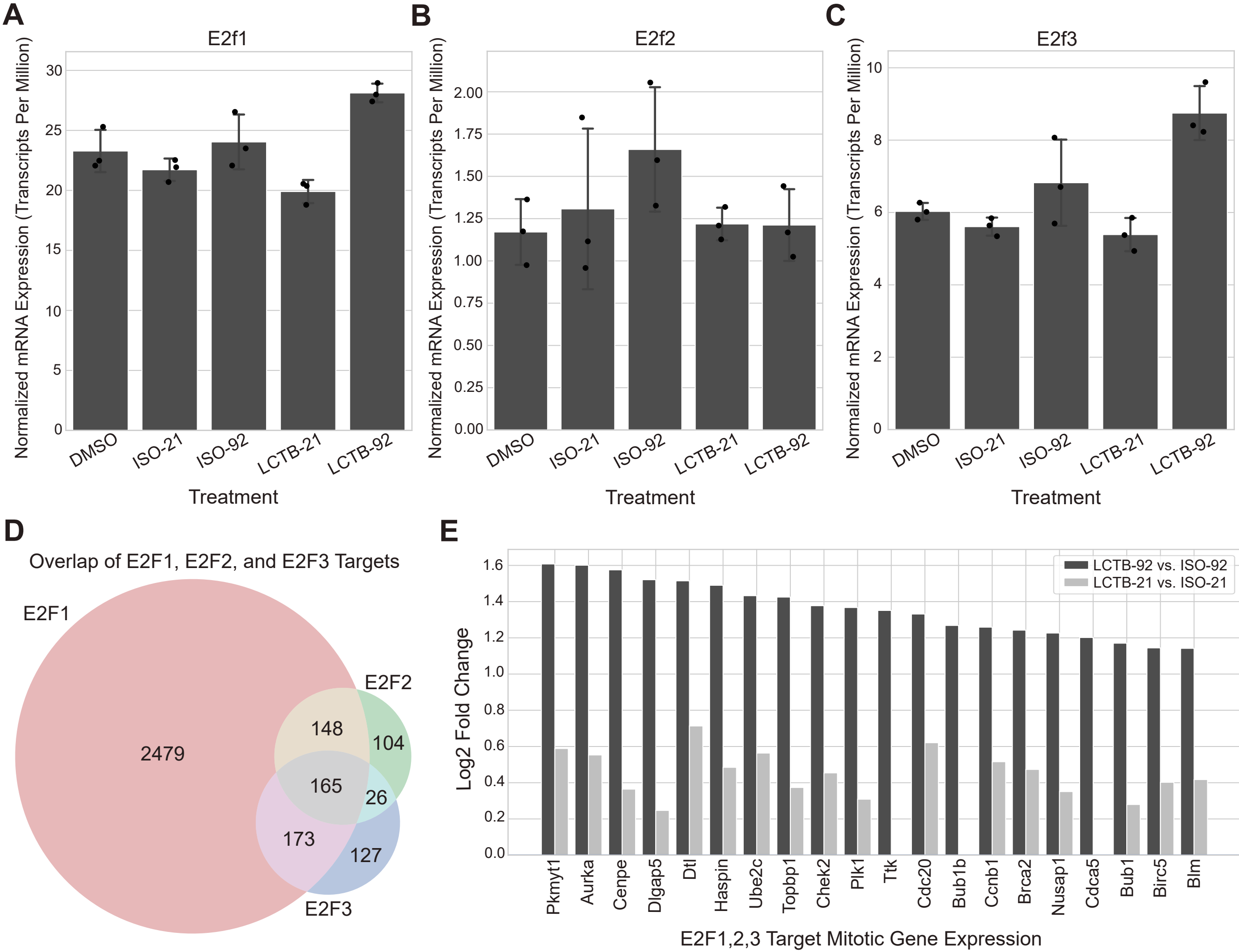
A
E2f1
Normalized mRNA Expression (Transcripts Per Million)
Treatment
DMSO
ISO-21
ISO-92
LCTB-21
LCTB-92
B
E2f2
C
E2f3
D
Overlap of E2F1, E2F2, and E2F3 Targets
E2F1
E2F2
E2F3
2479
148
104
165
26
173
127
E
LCTB-92 vs. ISO-92
LCTB-21 vs. ISO-21
Log2 Fold Change
E2F1,2,3 Target Mitotic Gene Expression
Pkmyt1
Aurka
Cenpe
Dlgap5
Dtl
Haspin
Ube2c
Topbp1
Chek2
Plk1
Ttk
Cdc20
Bub1b
Ccnb1
Brca2
Nusap1
Cdca5
Bub1
Birc5
Blm

Figure S1: Increased E2F1/2/3 activity in drug-treated NRCMs. (A–C) Normalized mRNA expression of (A) E2F1, (B) E2F2, and (C) E2F3 across treatment groups in neonatal rat cardiomyocytes (NRCMs). (D) Venn diagram of predicted E2F target genes identified using ChEA3 (mean rank library). (E) Significant differentially expressed mitotic genes (as determined by GO term enrichment gene sets) that are predicted E2F targets in LCTB-92 or LCTB-21-treated NRCMs relative to respective isomer controls. Statistical significance for panels A–C was assessed using one-way ANOVA followed by post hoc pairwise comparisons to DMSO with Benjamini-Hochberg correction for multiple testing.
